# Minimally Invasive Valve Repair and Hybrid Treatment for Racemose Bronchial Artery Malformation With Coronary Arterial Inflow

**DOI:** 10.1093/icvts/ivag202

**Published:** 2026-07-20

**Authors:** Makoto Matsuura, Toshiaki Ito, Satoshi Ariumura, Motoki Nagatsuka

**Affiliations:** Department of Cardiovascular Surgery, Shonan Kamakura General Hospital, Kanagawa 247-8533, Japan; Department of Cardiovascular Surgery, Shonan Kamakura General Hospital, Kanagawa 247-8533, Japan; Department of Cardiovascular Surgery, Shonan Kamakura General Hospital, Kanagawa 247-8533, Japan; Department of Cardiovascular Surgery, Shonan Kamakura General Hospital, Kanagawa 247-8533, Japan

**Keywords:** racemose haemangioma, coronary arterial inflow, hybrid strategy, minimally invasive cardiac surgery, collateral circulation

## Abstract

Racemose haemangioma of the bronchial artery is a rare vascular malformation characterized by abnormal communication between the bronchial and pulmonary vasculature. Coronary arterial inflow may further complicate minimally invasive cardiac surgery because excessive collateral flow can impair myocardial protection and increase intracardiac return during cardiac arrest. We report a case of atrial functional mitral regurgitation and tricuspid regurgitation associated with racemose bronchial artery malformation with coronary arterial inflow from the left circumflex artery. Preoperative embolization of systemic feeders from the bronchial and left subclavian arteries was followed by minimally invasive valve repair and surgical ligation of the coronary inflow vessel. The postoperative course was uneventful. A staged hybrid strategy may be effective for managing racemose bronchial artery malformation with coronary arterial inflow.

## INTRODUCTION

Racemose haemangioma of the bronchial artery is a rare vascular malformation characterized by abnormal communication between the bronchial and pulmonary vasculature.^1^ Excessive collateral return may impair myocardial protection and cause cardiac distention during cardiac arrest, particularly in cases with coronary arterial inflow.^2^

We report a rare case of racemose bronchial artery malformation with coronary arterial inflow managed with a staged hybrid approach and concomitant minimally invasive valve repair.

## CASE REPORT

A 78-year-old male presented with severe mitral and tricuspid regurgitation. Echocardiography demonstrated atrial functional mitral regurgitation with annular dilatation.

**Figure 1.**
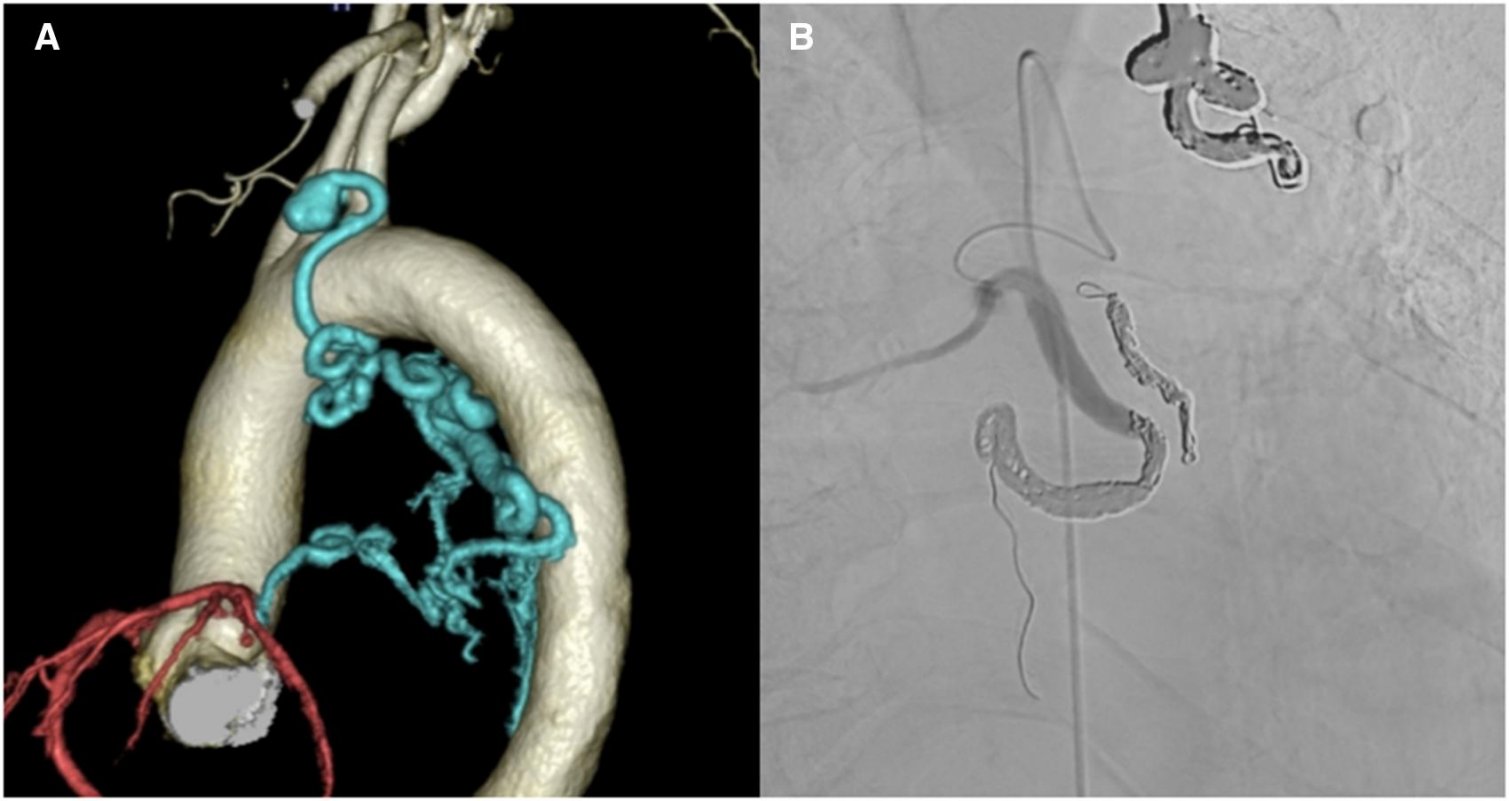
Preoperative Imaging. (A) CT angiography showing systemic-to-pulmonary artery feeders from the bronchial and left subclavian arteries. (B) Transarterial coil embolization of the systemic feeders.

Computed tomography and coronary angiography revealed a coronary–pulmonary artery fistula from the left circumflex artery and multiple systemic-to-pulmonary artery fistulas from the bronchial and left subclavian arteries (**Figure 1A**). The bronchial artery was markedly dilated and tortuous, findings consistent with racemose haemangioma of the bronchial artery.

Given the high-flow collateral supply and risk of cardioplegic runoff and cardiac distention, a staged strategy was adopted. Transarterial coil embolization of the bronchial and left subclavian feeders was performed close to the pulmonary parenchyma to reduce shunt flow (**Figure 1B**).

Surgery was performed on the following day after embolization. Cardiopulmonary bypass was established via the left femoral artery (20 Fr) and left femoral vein (23/25 Fr 2-stage cannula). A minimally invasive approach was performed through 3 ports in the right third, fourth, and fifth intercostal spaces. The inferior vena cava was snared. After aortic cross-clamping and antegrade cardioplegia, the left atrium was opened for ventricular decompression.

The transverse sinus was exposed, and an abnormal vessel originating from the left circumflex artery and running along the roof of the left atrium towards the pulmonary artery was identified (**Video 1**). The fistulous vessel was ligated at its origin using two 3–0 non-absorbable sutures. Additional cardioplegia was subsequently administered to ensure adequate myocardial protection after ligation of the fistula.

Mitral valve repair was performed for atrial functional mitral regurgitation. Secondary chordae of the anterior leaflet were divided, and a 32-mm saddle-shaped rigid annuloplasty ring (St. Jude Medical, Abbott) was implanted.

The left atrial appendage was resected using a linear stapler.

The superior vena cava was snared, and the right atrium was opened.

Notably, blood return to the right heart remained greater than that typically observed during conventional cardiac procedures, even after preoperative embolization (**Video 2**).

Tricuspid valve repair was performed using a 29-mm Tailor Annuloplasty Ring (Abbott), avoiding suture placement in the conduction system area.

**Figure 2.**
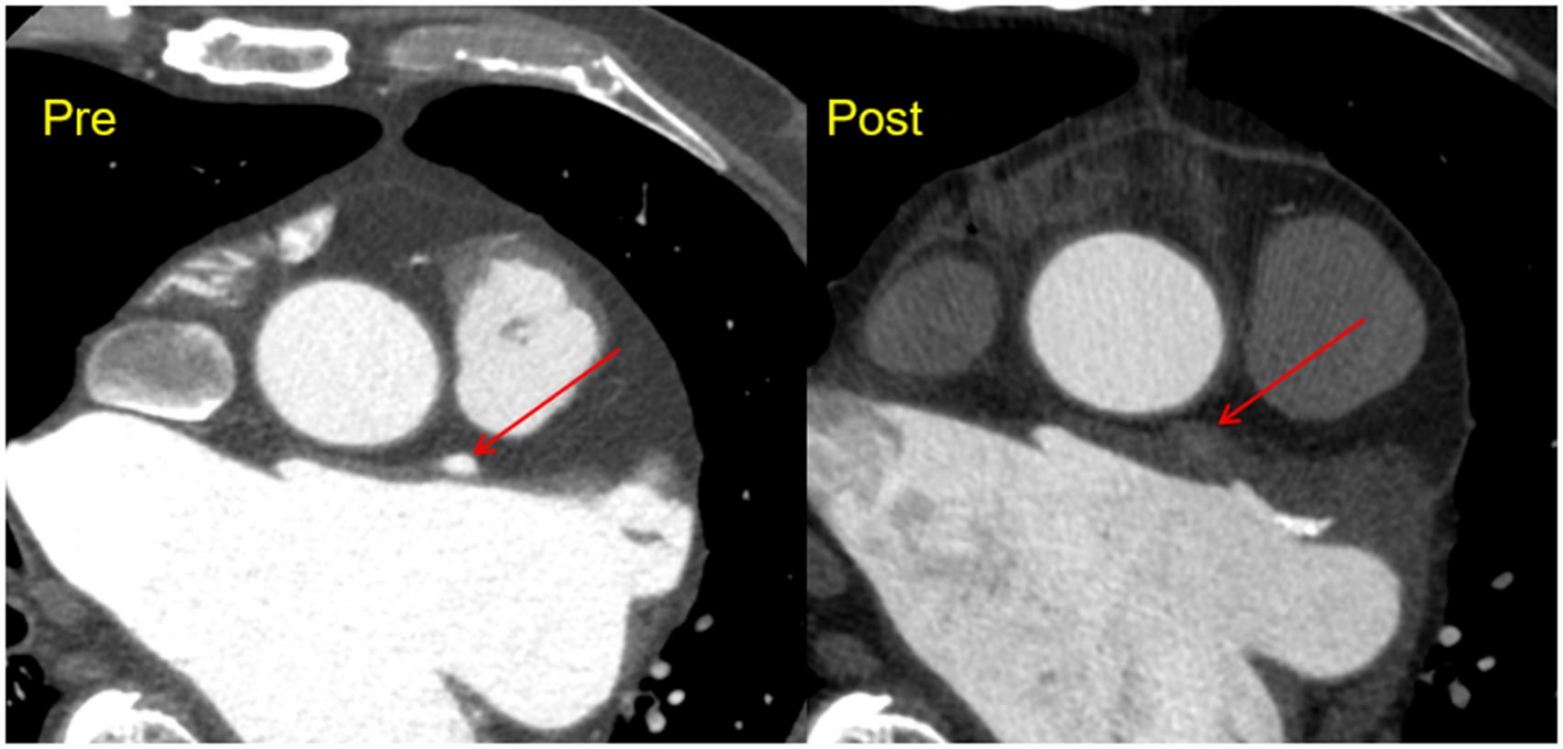
Postoperative Findings. Preoperative and postoperative CT images demonstrating elimination of the fistulous communication after hybrid treatment.

The patient was weaned from cardiopulmonary bypass without difficulty. The cardiopulmonary bypass time was 141 min, and the aortic cross-clamp time was 94 min. The postoperative course was uneventful. Postoperative transthoracic echocardiography demonstrated no residual mitral regurgitation and mild tricuspid regurgitation. Follow-up computed tomography confirmed elimination of all fistulous communications (**Figure 2**).

## DISCUSSION

This case highlights the haemodynamic challenges of racemose bronchial artery malformation with coronary arterial inflow during minimally invasive cardiac surgery.

Racemose haemangioma of the bronchial artery is characterized by abnormal communication between the bronchial and pulmonary vasculature.^3^ It may present with haemoptysis and potentially increase the risk of intraoperative pulmonary haemorrhage during minimally invasive surgery because of fragile collateral vessels.^3^ Additional coronary inflow may further increase collateral return during cardiac arrest.

Minimally invasive coronary artery fistula ligation has been reported in selected patients.^4^ However, extensive collateral circulation may complicate myocardial protection and increase intracardiac return during cardiac arrest.^1^^,^^2^^,^^5^ In our case, persistent oxygenated blood return into the right heart was observed despite preoperative embolization, suggesting substantial residual collateral inflow.

Preoperative embolization of non-coronary feeders reduced collateral flow and likely prevented more severe cardiac distention. The coronary fistula was suitable for direct ligation through the transverse sinus, whereas the systemic feeders were not readily accessible through the right mini-thoracotomy approach. Accordingly, a hybrid strategy combining surgical ligation and embolization was adopted.

Both valve repair and fistula treatment were safely completed through a minimally invasive approach.

## CONCLUSION

A staged hybrid strategy combining embolization and minimally invasive surgery may be effective for managing racemose bronchial artery malformation with coronary arterial inflow and complex collateral circulation.

## Data Availability

The data that support the findings of the study are available from the corresponding author upon reasonable request.
